# Diagnostic retinal biopsy in the management of secondary non-CNS vitreoretinal lymphoma masquerading as viral retinitis: a case report

**DOI:** 10.1186/s40942-021-00327-3

**Published:** 2021-10-11

**Authors:** Aditya Rali, Lucy T. Xu, Caroline Craven, Jonathon B. Cohen, Steven Yeh, Hans E. Grossniklaus, Ghazala D. O’Keefe

**Affiliations:** 1 Department of Ophthalmology, Emory University School of Medicine, Atlanta, GA USA; 2 Department of Hematology and Medical Oncology, Emory University School of Medicine, Atlanta, GA USA; 3 Truhlsen Eye Institute, University of Nebraska Medical Center, Omaha, NE USA; 4 Uveitis and Retinal Disease, Emory Eye Center, Emory University School of Medicine, Atlanta, USA

**Keywords:** Lymphoma, Large B-Cell, Diffuse, Intraocular lymphoma, Retina, Biopsy, Vitreoretinal surgery, Vitrectomy, Retinitis

## Abstract

**Background:**

Intraocular lymphoma accounts for fewer than 1% of intraocular tumors. When the posterior segment is involved, it can be further classified as vitreoretinal or choroidal lymphoma. Vitreoretinal lymphoma (VRL) can rarely masquerade as an infectious retinitis making diagnosis and management challenging.

**Results:**

A 73-year-old woman with a history of non-central nervous system (CNS) involving diffuse large B-cell lymphoma (DLBCL) was referred for worsening blurry vision—visual acuity of count figures at 2 ft—in her right eye for 8 months. Dilated fundus examination of the right eye was significant for retinal whitening and dot-blot hemorrhages, which was concerning for a viral retinitis and guided initial management. Secondary intraocular lymphoma was also considered. The retinal disease continued to progress despite intravitreal and systemic antiviral therapy, and a diagnostic vitrectomy was inconclusive. A retinal biopsy was then performed, which showed DLBCL, confirming a diagnosis of secondary VRL. Three subsequent treatments with intravitreal methotrexate led to regression of the VRL.

**Conclusions:**

Our case highlights the utility of a retinal biopsy after an inconclusive diagnostic vitrectomy in a challenging scenario of VRL to establish a diagnosis and initiate successful treatment. A multidisciplinary team of providers was essential for diagnosis, comprehensive workup, medical and surgical management of the patient.

**Supplementary Information:**

The online version contains supplementary material available at 10.1186/s40942-021-00327-3.

## Background

Intraocular lymphoma accounts for fewer than 1% of intraocular tumors [1]. When the posterior segment is involved, it can be further classified as vitreoretinal or choroidal lymphoma. Previous case reports have highlighted that vitreoretinal lymphoma (VRL) can rarely masquerade as an infectious retinitis making diagnosis and management challenging [2–5]. We describe a case of secondary VRL in a patient with a history of diffuse large B-cell lymphoma (DLBCL) that mimicked a viral retinitis during the patient’s initial presentation. Retinal biopsy was eventually required to achieve a pathologic diagnosis that guided management with intravitreal methotrexate therapy.

## Case presentation

**Fig. 1:**
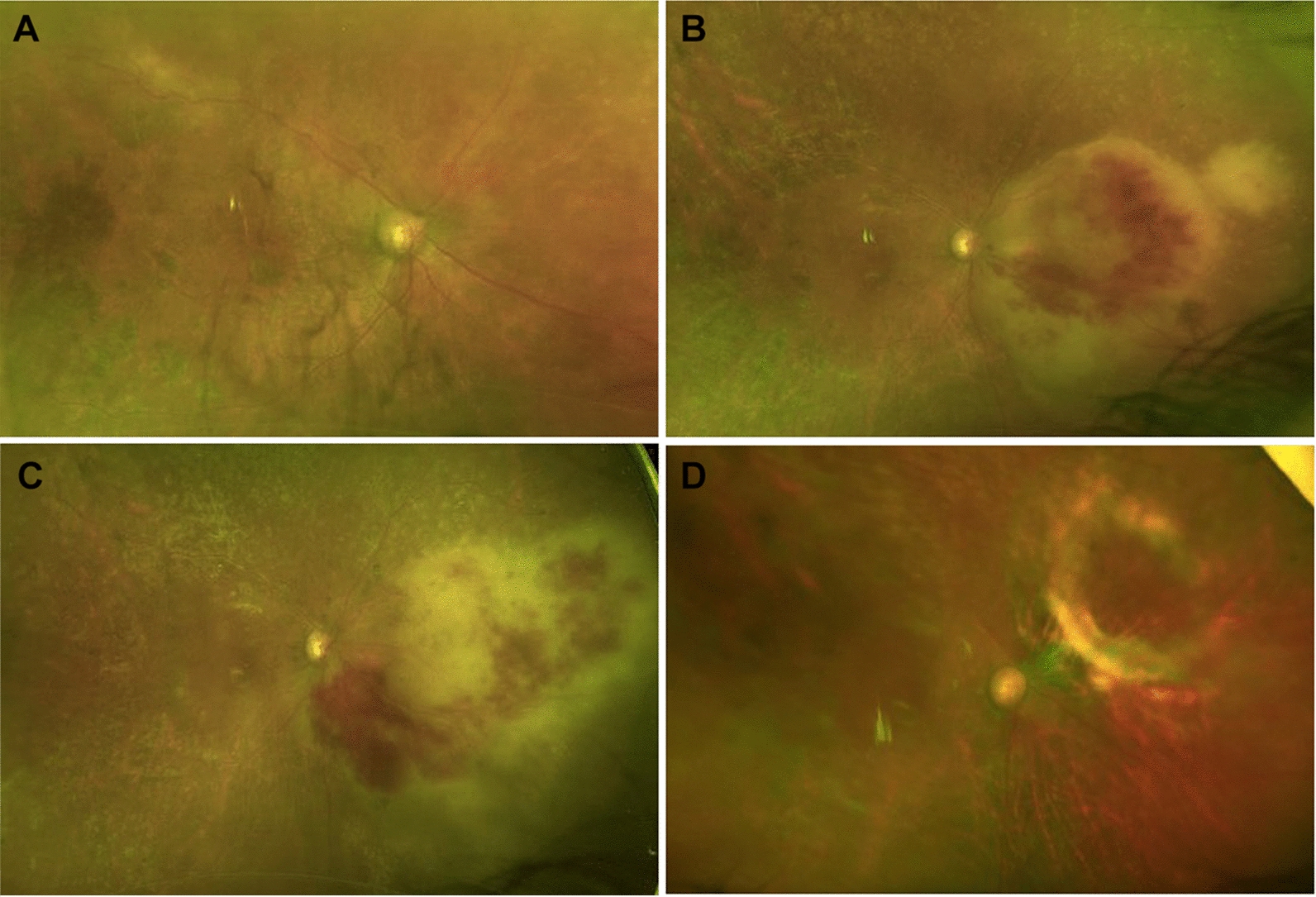
Fundus photograph at baseline shows mild vitreous haze with retinal pigment epithelial change and vascular sheathing (**A**). Clinical progression of retinitis was observed with a circular area of whitening and central hemorrhage (**B**). Additional progression with increased nasal whitening prompted retinal biopsy (**C**) with excellent resolution at four-month follow-up (**D**)

**Fig. 2:**
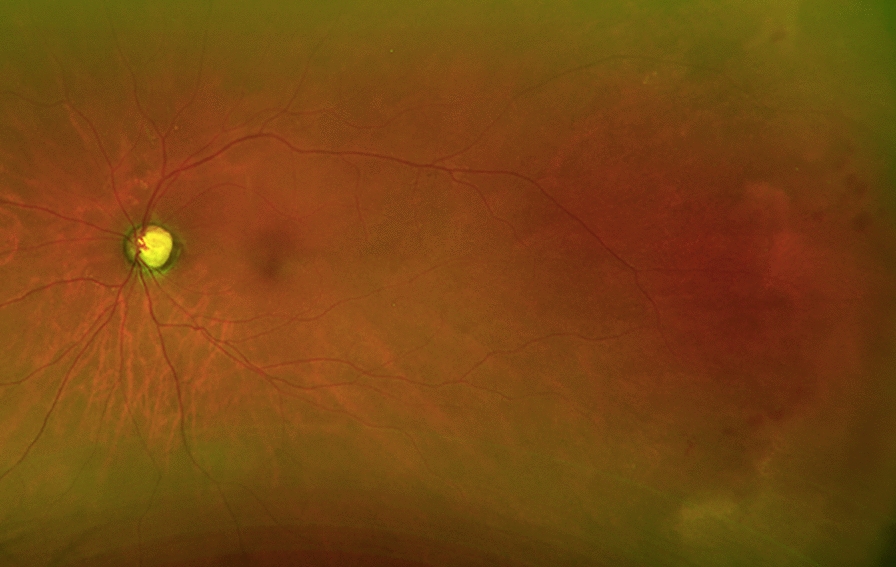
Fundus photograph of the left eye at initial presentation showing severely enlarged cup-to-disc and few temporal dot-blot hemorrhages

A 73-year-old woman with a history of non-central nervous system (CNS) involving DLBCL was referred for worsening blurry vision in her right eye for 8 months. Her DLBCL was initially diagnosed three years prior when she developed a neck mass and further imaging showed widespread adenopathy, splenomegaly and large right pleural effusion. More recently, she had been followed by an outside retina specialist and had received 6 intravitreal ganciclovir injections in the right eye along with oral valganciclovir for presumed CMV retinitis. Her ocular history was also remarkable for primary open angle glaucoma, affecting the left more than the right eye. Best corrected visual acuity was counting fingers at 2 ft in the right and 20/500 in left eye. The vision loss in the left eye was secondary to severe primary open angle glaucoma. Anterior segment examination of the right eye showed inferior keratic precipitates and trace cell and flare, while the left eye was unremarkable. Dilated fundus examination of the right eye showed 1+ anterior vitreous cell, mild vitreous haze, 0.5 cup-to-disc ratio with sharp rims, scattered areas of retinal whitening, most pronounced along vasculature, and diffuse dot blot hemorrhages and chorioretinal scars peripherally (Fig. 1a). Dilated fundus examination of the left eye showed 0.9 cup-to-disc with pallor and few temporal dot blot hemes (Fig. 2). Due to the ongoing suspicion for infectious retinitis, she was initially treated with intravitreal foscarnet and continued on oral valganciclovir. An anterior chamber paracentesis was negative for cytomegalovirus, herpes simplex virus, varicella zoster virus and toxoplasmosis by polymerase chain reaction testing.

Secondary intraocular lymphoma was also considered due to subjective visual changes following the identification of a local gastro-hepatic recurrence of DLBCL 3 months prior to presentation to our service. Following the negative aqueous humor testing for herpetic viruses, a diagnostic vitrectomy was performed. Bacterial cultures and viral PCRs were negative, and cytopathology revealed non-specific chronic inflammation with proteinaceous material. Her visual acuity subjectively declined with progressive disease including new areas of retinal whitening and intraretinal hemorrhages (Fig. 1b, c).

**Fig. 3:**
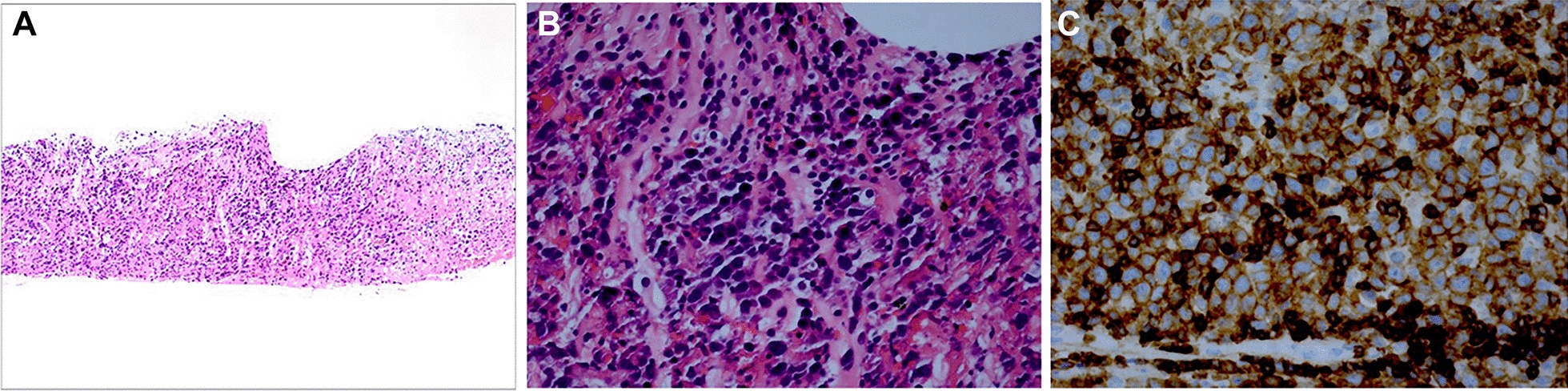
**A** The retina is infiltrated with numerous lymphocytes (hematoxylin and eosin ×25). **B** The lymphocytic infiltrate includes small lymphocytes and larger lymphocytes with higher nuclear to cytoplasmic ratios (hematoxylin and eosin ×100). **C** Immunohistochemical stains are positive for CD20 in the lymphocytes (peroxidase anti-peroxidase ×100)

The patient initially deferred further surgery, but due to clinical progression, a diagnostic retinal biopsy was performed (Additional file 1: Video S1). During standard 23-gauge vitrectomy, endodiathermy was used to mark the retinal biopsy site in the superonasal quadrant involving both normal and abnormal-appearing retina. Retinal scissors were used to incise the retina along the diathermized outline and internal limiting membrane (ILM) forceps were used to elevate the retinal tissue from the RPE. The superotemporal sclerotomy site was enlarged so the retinal biopsy could be extracted. Endolaser was applied to the edge of the retinal biopsy site followed by air-fluid exchange and 14% C3F8 instillation. Pathology was consistent with diffuse large B-cell lymphoma, confirming a diagnosis of secondary VRL (Fig. 3). Systemic workup, including a PET scan and MRI brain/orbits, did not show evidence of systemic DLBCL recurrence. The patient was subsequently treated with monthly intravitreal methotrexate 400 mcg/0.1 mL for 3 months with regression of the VRL and no evidence of recurrence over the 6-month follow-up period (Fig. 1d).

## Discussion and conclusions

While non-CNS lymphoma has a much stronger predilection for the choroid than the retina [2, 6], our case was notable given its primary involvement of the retina. The clinical disease findings mimicked an infectious retinitis, making the diagnosis particularly challenging. Polymerase chain reaction testing is highly sensitive for DNA viruses [7] and negative PCR testing from the aqueous humor and vitreous obtained from the vitrectomy procedure raised our suspicion that the patient’s findings could indeed represent secondary lymphoma masquerading as an infectious retinitis. Previous reports have highlighted intraocular lymphoma masquerading as infectious retinitis, but only Say et al. utilized a retinal biopsy to confirm the diagnosis [3–6].

The retinal biopsy required close communication preoperatively with the Ophthalmic Pathology team and intraoperatively, we ensured meticulous hemostasis and controlled enlargement of the sclerotomy when the retinal biopsy was extracted. These steps were important to maintain visualization of the retinal biopsy and prevent the retinal tissue from falling posteriorly at the time of extraction. Given that there was no obvious subretinal fluid around the biopsy site, we also pre-placed endolaser prior to the air-fluid exchange in the event that the view became cloudy during the air-fluid exchange which could have precluded adequate endolaser.

In deciding on the medication regimen for the patient, our Retina and Uveitis service worked closely with the Hematology-Oncology service for the patient’s medical workup, which showed no evidence of active, systemic lymphoma. Thus, we elected to proceed with intravitreal methotrexate on a monthly basis with close monitoring of treatment response. We recently reviewed our experience with monthly intravitreal methotrexate injections and found that the majority of patients who received monthly intravitreal methotrexate injections avoided relapse and achieved partial or complete remission with a mean treatment duration of approximately 60 days [8]. Patients in that series also showed mean improvement in visual acuity [8]. In this case, monthly intravitreal methotrexate for a series of three doses led to disease regression at final follow-up. Indeed, prior case reports and series have successfully used methotrexate for intraocular lymphoma without serious adverse reactions although keratopathy has been reported [9, 10].

In summary, our case report describes a challenging case of secondary intraocular lymphoma that presented with clinical findings suggestive of viral retinitis. A retinal biopsy was essential to establish a pathologic diagnosis following an inconclusive diagnostic vitrectomy and informed ongoing patient management in conjunction with a multidisciplinary team of providers.

## Supplementary Information

**Additional file 1: Figure 1.**Fundus photograph at baseline shows mild vitreous haze with retinal pigment epithelial change and vascular sheathing (**A**). Clinical progression of retinitis was observed with a circular area of whitening and central hemorrhage (**B**). Additional progression with increased nasal whitening prompted retinal biopsy (**C**)with excellent resolution at four-month follow-up (**D**).

## Data Availability

Not applicable.

## Abbreviations

VRL: Vitreoretinal lymphoma
CNS: Central nervous system
DLBCL: Diffuse large B-cell lymphoma

## References

[1] Mochizuki M, Singh AD (2009). Epidemiology and clinical features of intraocular lymphoma. Ocul Immunol Inflamm..

[2] Reddy V, Winslow R, Cao JH, Robertson ZM, Chen B, Ufret-Vincenty RL. Vitreoretinal lymphoma, secondary to non-CNS systemic lymphoma, masquerading as an infectious retinitis. Am J Ophthalmol Case Rep. 2019;16. 10.1016/j.ajoc.2019.100545 PMC6711862 31468000

[3] Ryan ME, Shantha JG, Grossniklaus HE, Yeh S (2015). Secondary vitreoretinal lymphoma masquerading as acute retinal necrosis. Ophthalmic Surg Lasers Imaging Retina..

[4] Zloto O, Elkader AE, Fabian ID, Vishnevskia-Dai V (2015). Primary vitreoretinal lymphoma masquerading as refractory retinitis. Case Rep Ophthalmol..

[5] Say EA, Knupp CL, Gertsch KR, Chavala SH (2012). Metastatic B-cell lymphoma masquerading as infectious retinitis and vasculitis. Oncol Lett.

[6] Coupland SE, Damato B (2008). Understanding intraocular lymphomas. Clin Exp Ophthalmol..

[7] Harper TW, Miller D, Schiffman JC, Davis JL (2009). Polymerase chain reaction analysis of aqueous and vitreous specimens in the diagnosis of posterior segment infectious uveitis. Am J Ophthalmol.

[8] Anthony C, Bavinger C, Shantha J, Voloschin A, O’Keefe G, Grossniklaus H, Yeh S (2021). Clinical outcomes following intravitreal methotrexate therapy for vitreoretinal lymphoma. Invest Ophthalmol Vis Sci.

[9] Frenkel S, Hendler K, Siegal T, Shalom E, Peer J (2008). Intravitreal methotrexate for treating vitreoretinal lymphoma: 10 years of experience. Br J Ophthalmol..

[10] Zhou X, Zhou X, Shi H, Lai J, Wang Q, Li Y, Chen K, Li Q, Zhou Q, Cao X, Chen B, Xiao J. Reduced frequency of Intravitreal methotrexate injection lowers the risk of keratopathy in vitreoretinal lymphoma patients. BMC Ophthalmol. 2020;20(1):189. 10.1186/s12886-020-01464-3. 10.1186/s12886-020-01464-3 PMC7216350 32397978

